# Biological organ ages associate with risk of chronic diseases in a community-based population

**DOI:** 10.1172/jci.insight.197304

**Published:** 2025-12-08

**Authors:** Celina S. Liu, Wan-Jin Yeo, Aditya Surapaneni, B. Gwen Windham, Hamilton S.-H. Oh, Anna Prizment, Sanaz Sedaghat, Pascal Schlosser, Eugene P. Rhee, Sushrut S. Waikar, Josef Coresh, Keenan A. Walker, Morgan E. Grams

**Affiliations:** 1 Division of Precision Medicine, Department of Medicine, New York University, New York, New York, USA.; 2 Department of Radiation Oncology, Department of Medicine, UCSF, San Francisco, California, USA.; 3 The MIND Center, University of Mississippi Medical Center, Jackson, Mississippi, USA.; 4 Department of Stem Cell Operations, Stanford University, Palo Alto, California, USA.; 5 Division of Laboratory Medicine and Pathology, Department of Medicine, and; 6 Division of Epidemiology & Community Health, School of Public Health, University of Minnesota, Minneapolis, Minnesota, USA.; 7 Department of Epidemiology, Johns Hopkins Bloomberg School of Public Health, Baltimore, Maryland, USA.; 8 Institute of Genetic Epidemiology, Medical Center – University of Freiburg, Faculty of Medicine, and; 9 Centre for Integrative Biological Signalling Studies (CIBSS), University of Freiburg, Freiburg, Germany.; 10 Nephrology Division and Endocrine Unit, Massachusetts General Hospital, Boston, Massachusetts, USA.; 11 Section of Nephrology, Department of Medicine, Boston Medical Center and Boston University Chobanian & Avedisian School of Medicine, Boston, Massachusetts, USA.; 12 Department of Population Health, and; 13 Optimal Aging Institute, New York University, New York, New York, USA.; 14 Laboratory of Behavioral Neuroscience, National Institute on Aging, Intramural Research Program, Baltimore, Maryland, USA.

**Keywords:** Aging, Cardiology, Biomarkers

## Abstract

The biological age of organs may better quantify risk for health deterioration compared with chronological age. We investigated organ-specific aging patterns in a community-based cohort and assessed the associations with adverse health outcomes. Biological ages of 11 organs were estimated for 11,757 participants of the Atherosclerosis Risk in Communities (ARIC) study (55.6% women, mean age, 57.1 years) using a circulating protein–based model. Older organ ages were significantly associated with related adverse outcomes, even after accounting for chronological age; for example, older arteries and hearts were associated with an increased risk for coronary heart disease (CHD; hazard ratio [HR] per 5-year-higher age gap, 1.22; 95% CI [1.13–1.31] and 1.16 [1.07–1.26], respectively, and older lungs with lung cancer (HR 1.12 [1.09–1.16]). Hierarchical agglomerative clustering based on organ ages revealed 3 patient phenotypes: those with older organs, normal/slightly older organs, and younger organs. The patients with older organs were at higher risk for cancer (HR 1.19; 95% CI [1.08–1.31]), death (HR 1.75 [1.64–1.86]), end-stage kidney disease (HR 6.12 [4.65–8.06]), CHD (HR 1.21 [1.06–1.38]), heart failure (HR 1.92 [1.73–2.13]), infection (HR 1.56 [1.44–1.68]), and stroke (HR 1.36 [1.16–1.61]). Proteomic organ aging signatures demonstrated significant associations with multiple adverse health outcomes and may be useful for health risk identification.

## Abstract 2

Organ ages, as estimated using levels of circulating proteins, are useful in predicting subsequent development of chronic disease.

## Graphical abstract

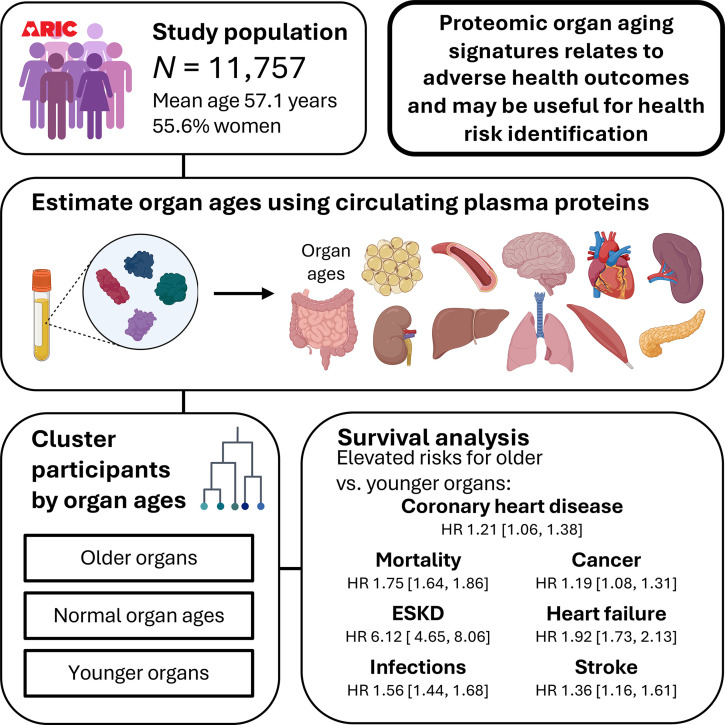

## Introduction

Aging is associated with an increased risk of multiple chronic diseases due to the physiological changes that accompany the deterioration of cellular function (1–4). Improvements in medicine and technology have dramatically increased global life expectancy; the Global Burden of Disease 2021 Study (GBD 2021) estimated that from 1950 to 2021, the global life expectancy increased from 49.0 years to 71.7 years, but with a corresponding increase in the population burden of chronic diseases (5). In addition, survival remains highly heterogeneous, with some individuals living well into their 90s with few comorbidities and others experiencing early mortality despite advances in care. Studies of the underlying mechanisms and pathways of aging may facilitate the development of beneficial therapies to promote resilience in the aging population.

The concept of biological age has been put forward as an explanation of the variability in life expectancy. The central idea is that organs age differently in different individuals, and these ages may provide health risk information beyond chronological age (6–10). For example, older brain ages estimated from MRI images have been associated with dementia within participants of the Knight Alzheimer’s Disease Research Center (Knight-ADRC) cohort (11). Motivated by the variation in age-related health trajectories across individuals, as well as significant associations between certain organ-specific proteins in the blood with age and age-related diseases, Oh and colleagues developed proteomic models to estimate individual organ ages using protein sets with enriched expression in each organ system (4-fold higher compared with other organs) (12). These results provided face validity to the supposition that estimated biological and organ ages may encode information about health beyond chronological age. However, the set of organ-specific outcomes examined and the follow-up time for these outcomes has thus far been limited.

To extend the work evaluating biological age and adverse outcomes, we applied the proteomic-based aging models developed by Oh et al. (12) to estimate organ-specific ages for participants in the Atherosclerosis Risk in Communities (ARIC) study, a community-based, prospective cohort followed for multiple outcomes, including mortality, cancer incidence, coronary heart disease (CHD), end-stage kidney disease (ESKD), heart failure (HF), infection, and stroke. Organ age gaps were estimated as the difference between estimated biological age and chronological age and were evaluated individually as well as a part of overall aging patterns, identified via hierarchical agglomerative clustering on participants’ estimated organ age gap. Longitudinal associations between the estimated age gaps, aging patterns, and the adverse outcomes were assessed over 26 years of follow-up.

## Results

### Study population characteristics.

Among 11,757 participants (mean age, 57.1 years [SD, 5.7], 55.6% women, 23.1% identified as Black race), the mean SBP was 122 mmHg (SD, 19), mean BMI was 27.9 (SD, 5.3), mean estimated glomerular filtration rate based on creatinine and cystatin C (eGFR_Cr-Cys_) was 98 mL/min/1.73 m^2^ (SD, 17), and mean total cholesterol level was 210 mg/dL (SD, 40). A total of 15.1% had diabetes, 33.3% were on anti-hypertensive (anti-HTN) medications, 2.0% had prevalent stroke, 6.5% had prevalent CHD, 5.1% had prevalent HF, and 22.3% and 33.3% were current and former smokers, respectively (Table 1).

### Prevalence of individual organ age gaps.

**Figure 1:**
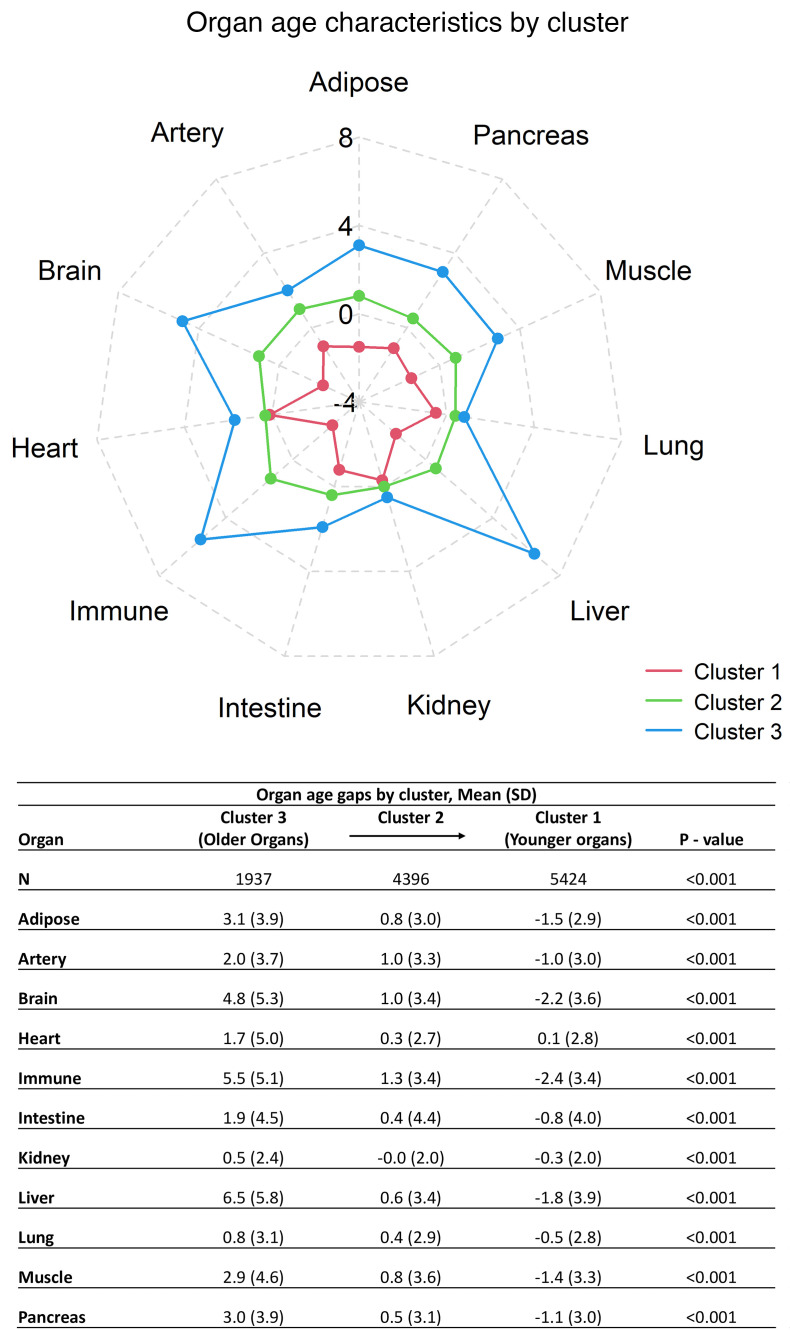
Estimated organ age gaps. Spider plot of patient clusters formed through hierarchical agglomeration using estimated organ age gaps and table showing the mean estimated organ age gaps for each cluster.

The most prevalent older organ, defined as an estimated organ age of more than 5 years older than their chronological age, was liver (15.2%), followed by immune system (13.9%) and brain (12.2%). Hierarchical clustering identified 3 distinct phenotypes based on organ age. The clusters corresponded to groups with generally older organs, normal/slightly older organs, and younger organs (Figure 1). The 3 groups had significant differences in the prevalence of diastolic blood pressure, diabetes, hypertension, stroke, CHD, HF, eGFR, total cholesterol, smoking status, anti-HTN medication status, and antidiabetic medication status, as well as for all organ age gaps (*P* < 0.001), indicating unique clinical and organ aging signatures (Table 2). For the cluster corresponding to older organs, the mean liver age gap (6.5 [SD 5.8] years) was noticeably higher than the age gaps of other organs, whereas the other 2 clusters corresponding to normal and younger organs did not exhibit specificity toward any organ age gap.

**Figure 2:**
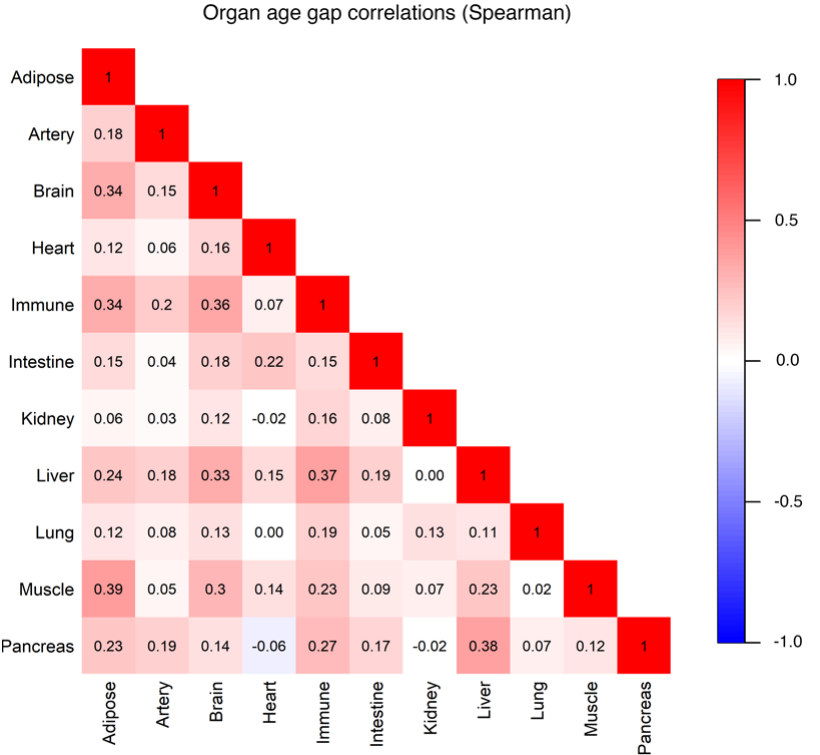
Inter-organ age gap. Estimated organ age less chronological age, using Spearman’s correlations.

The correlations of organ age gaps were moderate to low, with the highest between adipose and muscle (Spearman’s correlation = 0.39). Most correlations were positive (Figure 2).

### Associations of organ age gaps with incident adverse outcomes.

**Figure 3:**
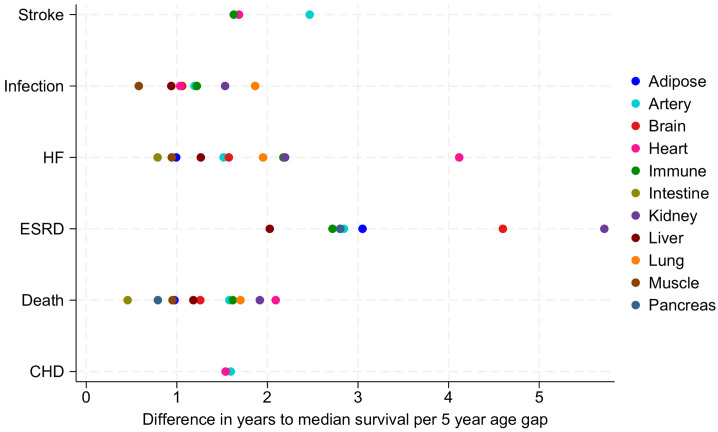
Difference in event-free survival time for older estimated organ gaps, per 5 years. Only statistically significant associations are displayed; there were no statistically significant associations with cancer. There were 1176 cases of stroke, 4859 hospitalized infections, 2596 cases of heart failure, 329 cases of end-stage kidney failure, 6975 deaths, 1928 cases of chronic heart disease, and 3526 cases of cancer over a mean follow-up of 26 years.

Over a mean follow-up of 26 years, older organ age gaps, a continuous measure of the estimated organ age less the chronological age, were associated with higher risks for several adverse health outcomes (Figure 3). For example, older arteries and older hearts were associated with an increased risk for CHD (hazard ratio [HR] 1.22; 95% CI [1.13–1.31] and 1.16 [1.07–1.26], respectively, per 5-year-higher organ age gap). Most outcomes were associated with 2 or more organ ages. The strongest associations were found between kidney (HR 2.26 [1.73–2.96]), brain (HR 1.78 [1.57–2.02]), adipose (HR 1.54 [1.29–1.84]), and muscle biological age (HR 1.52 [1.32–1.74]) and ESKD, and between heart biological age and HF (HR 1.62 [1.38–1.89]). None of the organ age gaps were associated with the incidence of total cancer. When considering individual cancer types as outcomes, older lung biological age was found to be a significant risk factor for lung cancer (HR 1.12 [1.09–1.16], per 5-year-higher age gap).

**Figure 4:**
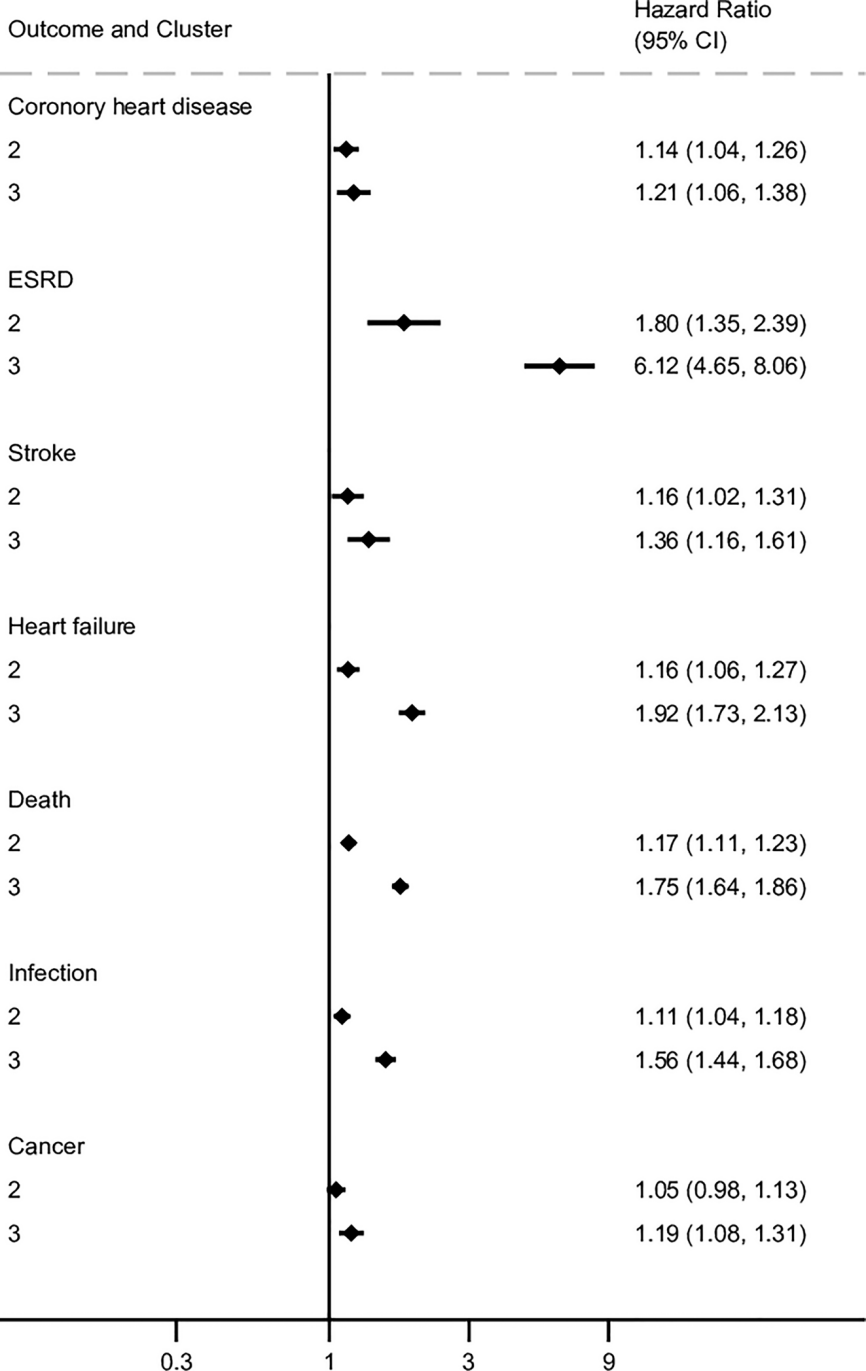
Forest plot showing the hazard ratios for older organs (Cluster 3) and normal-aged organs (Cluster 2) relative to younger organs (Cluster 1, reference) toward adverse outcomes. Older and normal organs were at higher risks for the adverse outcomes relative to younger organs.

Patient clusters related to organ age were also associated with adverse outcomes. Relative to the cluster corresponding to having younger organs, the clusters for older or normal organ ages were at higher risks for most adverse outcomes. For example, the oldest cluster was associated with higher risks of cancer (HR 1.19, 95% CI [1.08–1.31]), death (HR 1.75 [1.64–1.86]), ESKD (HR 6.12 [4.65–8.06]), HF (HR 1.92 [1.73–2.13]), infection (HR 1.56 [1.44–1.68]), and stroke (HR 1.36 [1.16–1.61]) (Figure 4).

### Pathways and proteins associated with organ clusters of interest.

After adjustment for demographics, proteins positively associated with the youngest organ cluster (cluster 1) included 6-phosphogluconate dehydrogenase (PGD), rho guanine nucleotide exchange factor 10 (ARHGEF10), cadherin-3 (CDH3), and the sodium/potassium-transporting ATPase subunit β-1 (ATP1B1) (all *P* < 0.001), with upregulation of the ubiquitin-like protein ligase activity and glycolysis/gluconeogenesis pathways (Supplemental Table 1; supplemental material available online with this article; https://doi.org/10.1172/jci.insight.197304DS1).

In contrast, proteins that showed the strongest positive associations with the older organ cluster (cluster 3) were tumor necrosis factor receptor superfamily 1 (TNFR1), netrin receptor UNC5B (UNC5B), WAP four-sulfide core domain protein 2 (HE4), and ephrin-B2 (EFNB2) (all *P* < 0.001). Pathway enrichment analyses demonstrated upregulation of pathways involving neutrophil degranulation (Supplemental Table 2).

## Discussion

In this large community-based study, we confirm the utility of organ age gaps estimated using a proteomic-based organ-specific aging model, extending findings to new adverse outcomes, a longer follow-up, and the identification of new phenotypes. Organ ages were associated not only with their corresponding outcome (e.g., kidney age with ESKD) but also with other outcomes, suggesting inter-organ dependencies for health. Although individual organ ages were only weakly correlated, clustering participants via minimizing intra-cluster organ age gap variances revealed an inherent hierarchy corresponding to having older, younger, and normal estimated ages across all organs. The cluster corresponding to the oldest organ ages was found to have the highest risk for cancer, mortality, ESKD, HF, infection, and stroke. These results suggest that protein-based age estimates may be a useful tool for health risk identification and explain in part the heterogeneity in survival among an older population.

The observation that more than one organ age was significantly associated with each outcome is consistent with the concept of inter-organ communication playing a role in biological aging and health. Age gaps of multiple organs, including the kidney, brain, adipose, and muscle had particularly strong positive associations with ESKD. Previous studies have shown changes to brain structure and functional connectivity, as well as a decline in cognition, for patients with ESKD, but to our knowledge there are no studies suggesting that brain age is a risk factor for kidney function decline (13–17). It has also been suggested that the dysregulation of adiponectin levels contributes to chronic kidney disease (CKD) progression, and it is well known that patients with ESKD often exhibit sarcopenia (18–24). Our finding of the association between older lungs and lung cancer is interesting in the context of a previous study from Lu et al. that reported aging-associated genes could predict lung cancer prognosis (25). In our study, organ ages were estimated using proteins associated with genes determined to be more highly expressed at the organ of interest, but it seems biologically plausible that aging-associated reductions in lung repair and maintenance might predispose to malignancy. On the other hand, an alternative hypothesis might posit that organs age separately yet in parallel, resulting in consistent associations with adverse outcomes.

A priori, we had expected moderate-to-strong inter-organ age gap correlations, hypothesizing that inter-organ communication regulates metabolic homeostasis and aging through complex signaling pathways (10, 12, 26–30). We did see a strong positive correlation between muscle and adipose age gaps (0.40), a finding that is complementary to studies on sarcopenic obesity suggesting adipokine- and myokine-mediated crosstalk between adipose and muscle tissues (31–36). Senescent cell accumulation, which has been shown to occur in multiple organs, might also be expected to contribute to positive inter-organ age gap correlations (37, 38). In contrast, we observed mostly moderate to low correlations of organ age gaps. One reason might be that the ARIC population was generally young at the time of organ age gap assessment (mean age 57.1 years). Diseases or conditions that may contribute to a strong pairwise age gap correlation between 2 organs such as sarcopenic obesity may not be prevalent enough to result in notable correlation signals.

Hierarchical clustering of the organ age gaps did demonstrate 3 distinct clusters, with patterns stratifying participants to have older, normal, or younger overall organ ages. These results differed from Oh et al.’s study, where k-means clustering resulted in clusters corresponding to extreme aging for each specific organ, with one that corresponded to extreme aging in multiple organs (12). The differences in each of our 3 clusters’ risks for cancer, CHD, mortality, ESKD, HF, infections, and stroke suggest the utility of clustering by organ age gaps, allowing participants to be stratified into different risk categories for adverse health outcomes. Prior studies have also found positive associations between older organ ages and worse health outcomes, especially with related diseases (26). Phenotyping through organ age gap clusters may allow for earlier disease screening, diagnosis, and prevention for higher-risk populations.

This study has multiple strengths, including the large sample size and the broad spectrum of outcomes that have been objectively defined and augmented with state/national registries. One limitation is the limited racial demographic, since only Black and White race categories were included, and the inclusion of only 4 US communities. This may reduce the generalizability of results to other populations. Another limitation concerns the validity of ascribing our conventional understanding of aging to the organ ages estimated using proteomic data, although its numerous intuitive associations with adverse outcomes indicate that information regarding health risk is being encoded. SomaLogic protein quantifications may have biases and do not yet quantify the entire proteome exhaustively. Thus, current estimates of organ ages may be improved if there exist relevant but yet unquantified proteins, or if proteomic measurements were drawn directly from relevant tissues (39). Finally, the assignment of a protein to a single organ may be overly simplistic given the widespread expression of many proteins. Despite these limitations, omics tools, particularly proteomics, have been shown to have great potential for understanding biological aging (40). Aging models might be further improved by incorporating DNA methylation or environmental/lifestyle influences, or by including changes in the proteome over time (6, 41).

In summary, our results support the concept that biological organ aging can be quantified and relates to multiple adverse outcomes. Biological organ ages estimated with aging biomarkers informed by epigenetic and omics studies may further our ability to identify health risk and prevent disease.

## Methods

### Sex as a biological variable.

Our study included both male and female participants from the ARIC cohort. Analyses included sex as a covariate, and results are reported for the full sample. The findings are expected to be relevant for both sexes.

### Study population.

The ARIC study is an ongoing community-based cohort study that enrolled 15,792 participants ages 45–65 years (baseline exam 1987–1989) from 4 different US communities (Forsyth County, North Carolina; Jackson, Mississippi; Minneapolis, Minnesota; and Washington County, Maryland) (42). The primary objectives of ARIC were to investigate risk factors, prevalence, and incidence of cardiovascular diseases.

We included participants who attended ARIC visit 2 (1990–1992), which was selected as the first visit in which proteomic profiling was performed. Participants were excluded if they did not identify as being in the Black or White race categories (*N* = 41), had missing proteomic data (*N* = 1651), had protein measurements that did not pass quality control (*N* = 126), or whose proteomic measurements were identified as outliers (first 10 principal components of proteomic measurements deviated by more than 5 SD; *N* = 53). The final number of participants included was 11,757.

### Protein measurement, preprocessing, and classification.

Blood samples were collected in ethylenediaminetetraacetic acid tubes, centrifuged, and the plasma was frozen at –80°C. In 2021, these samples were thawed and proteomic profiling was conducted using slow off-rate modified aptamer–based (SOMAmer-based) capture assays (SomaScan assay version 4.0, 5K; SomaLogic) (43, 44). The plasma protein measurements were calibrated and normalized by SomaScan using adaptive normalization by maximum likelihood (ANML) to account for assay and batch biases. A total of 5284 proteins were quantified. After quality control, excluding aptamers with low variance or reproducibility and those binding to non-protein targets or contaminants, proteins were log_2_-transformed to address skewed distributions, and winsorized when levels were more than 5 SD from the individual protein mean. The split-sample reliability coefficient was 0.94. Previous work has evaluated the identity of the proteins through protein quantitative trait loci (pQTLs) and cross validation with other proteomic platforms, including multiplexed measurements (45–48).

Oh et al.’s classified organ-specific proteins based on the definition proposed by the Human Protein Atlas, with mutual exclusivity, if there was a 4-fold or higher expression of the corresponding gene(s) in any tissue of that organ relative to tissues of other organs according to the Gene Tissue Expression Atlas (GTEx) tissue bulk RNA-seq database. They then developed models for organ-specific ages using these proteins as predictors and chronological age as the outcome (12, 43). Proteins included for each organ age model are listed in Supplemental Table 3.

### Aging model, definitions, and clustering.

The aging models predicting organ-specific ages developed by Oh et al. were trained as a bagged ensemble of least absolute shrinkage and selection operator (LASSO) models for 11 organs (adipose tissue, artery, brain, heart, immune tissue, intestine, kidney, liver, lung, muscle, and pancreas); while not an organ, the “immune organ” was defined with gene expression in blood and spleen tissues (12). These models used the identified organ-specific proteins as the training input (https://github.com/hamiltonoh/organage). In brief, the previously published model weights were applied to the ARIC cohort to determine predicted organ ages. These estimates were then calibrated to the ARIC population by fitting a locally estimated scatterplot smoothing (LOWESS) curve predicted on chronological age, and the residual was labeled as the “organ age gap” for each individual participant. Values greater than 5 years were considered large organ age gaps: younger organ age was defined as biological age more than 5 years younger than chronological age and older organ age was defined as biological age more than 5 years older than chronological age.

### Definitions of outcomes.

The outcomes of interest were incident cancer, CHD, mortality, ESKD, HF, infection, and stroke. Cancer incidence was defined as any one of bladder, brain, breast, cervical, colorectal, head and neck, kidney, liver, lung, multiple myeloma, pancreas, prostate, or thyroid cancer, determined via linkage to cancer registries in Minnesota, North Carolina, Maryland, and Mississippi, and augmented with information obtained from participants, hospital discharge summary codes (see Supplemental Table 4), and medical records (49–51). Mortality was determined from follow-up phone calls or the National Death Index, and ESKD incidence was determined from US Renal Data System (USRDS) registry data linked to the study (52). CHD was defined as adjudicated definite or probable myocardial infarction (MI), fatal coronary event, cardiac revascularization procedure(s), or silent MI from electrocardiogram data obtained at the visit (49, 53, 54). HF and infections were determined from ICD-9-CM and ICD-10-CM hospitalization discharge codes (53, 55–57). Stroke was defined as physician-adjudicated intracerebral hemorrhage or definite, probable, or possible ischemic stroke from hospitalization records that were eligible for validation, including those with discharge codes as listed in Supplemental Table 4 (58). For analyses of each outcome, patients with prevalent disease at baseline were excluded.

### Covariates of interest.

Covariates included age, sex (male or female), race-study center (self-reported Black or White race), smoking status (current, former, or never smoked), estimated glomerular filtration rate (eGFR_Cr-Cys)_, diabetes, systolic blood pressure (SBP), CHD, HF, stroke, anti-HTN medication status, total cholesterol, and BMI. Age, sex, race, and smoking status were self-reported. Study center consisted of 4 categories: Forsyth County, North Carolina, Jackson, Mississippi, Minneapolis suburbs, Minnesota, and Washington County, Maryland. eGFR_Cr-Cys_ was calculated using the race-free 2021 CKD-EPI equation from serum creatinine and serum cystatin C levels, the former determined using a modified kinetic Jaffé method and the latter using the Gentian turbidimetric immunoassay (59). Diabetes was defined as either having a blood glucose level of 140 mg/dL or higher after a fasting period of 8 or more hours, a non-fasting blood glucose level of 200 mg/dL or higher, self-reported diagnosis of diabetes by a physician, or being on medication(s) for diabetes or high blood sugar. SBP and diastolic blood pressure (DBP) were defined with the mean of the latter 2 of 3 sitting blood pressure measurements, each taken 5 minutes apart without a change in posture. Anti-HTN medication status was reviewed by looking at pill bottles during health exams, and BMI (defined with participants’ height and weight) and total cholesterol were measured during the visit. Information on the proportion missing each covariate is provided in Supplemental Table 5.

### Statistics.

Categorical baseline characteristics were summarized using counts and proportions, and continuous characteristics were summarized using mean and SD.

Participants were categorized according to the presence of one or more “older organs,” and the combinations of participants with different “older” organs were enumerated using an UpSet plot. Inter-organ age gap correlations were assessed with pairwise Spearman correlations using continuous organ age gaps.

To identify organ aging signatures, participants were clustered by greedy minimization of intra-cluster organ age gap variances (Ward’s linkage method), revealing 3 distinct clusters. Participant characteristics for each cluster, as well as the mean and SD of individual organ age gaps, were reported. Differences in these characteristics across the 3 clusters was assessed either using χ^2^ tests for binary variables or analysis of variance (ANOVA) tests for continuous variables. Cox proportional hazards models were implemented to assess differences in risks for cancer, CHD, ESKD, HF, infection, stroke, and mortality by categorical organ age gap and organ age cluster. Participants were followed from visit 2 until the event occurred, death, withdrawal from the study, or administrative censoring on December 31, 2020. Cancer outcomes were censored on December 31, 2015. Each model was adjusted for baseline age, sex, race and study-center combinations, smoking status, eGFR_Cr-Cys_, diabetes, SBP, CHD, HF, stroke, anti-HTN medication status, total cholesterol, and BMI. Associations were determined to be significant using a Bonferroni correction for the number of comparisons (e.g., *P* < 0.05/77, where 77 = no. of organs × no. of diseases). To further investigate whether there were associations between individual organ-specific gaps with the corresponding cancer type, a similar analysis was used to assess longitudinal associations between index exam (visit 2) organ age gaps and each of the 13 different types of cancers considered, with significance being defined as having a *P* value of less than 0.05/13^11^ (<3.5 × 10^–4^). To evaluate the proteins associated with organ age cluster, we used a *P* value of less than 0.05/4955 proteins. The top 100 proteins were entered into the STRNG Core Data Resource to visualize protein-protein association networks (60). Gene set enrichment analyses were performed to evaluate for over-representation of members of predefined gene sets by Gene Ontology, KEGG, and Reactome. A Benjamini-Hochberg adjusted *P* value of less than 0.05 for gene set enrichment was considered statistically significant.

### Study approval.

All participants provided written informed consent, and the institutional review boards at each study site approved the study, and the ARIC study was reviewed and approved by the Johns Hopkins University Institutional Review Board.

### Data availability.

Underlying data, including proteomics data, are accessible through NIH’s BioLINCC and by dbGaP (phs000280.v3.p1) as per NIH sharing policy in a future data freeze. Pre-existing data access policies for each of the parent cohort studies specify that research data requests can be submitted to each steering committee; these will be promptly reviewed for confidentiality or intellectual property restrictions and will not unreasonably be refused. Please refer to the data sharing policies of these studies. Individual level patient or protein data may further be restricted by consent, confidentiality or privacy laws/considerations. These policies apply to both clinical and proteomic data. Values for all data points in graphs are reported in the Supporting Data Values file.

## Author contributions

CSL and WJY drafted the manuscript. AS and WJY performed data analysis. HSHO conducted foundational preliminary work. BGW, AP, SS, PS, EPR, SSW, JC, MEG, and KAW critically revised the manuscript for important intellectual content. MEG designed and supervised the project. CSL and WJY are co–first authors; author order was determined alphabetically.

## Funding support

This work is the result of NIH funding, in whole or in part, and is subject to the NIH Public Access Policy. Through acceptance of this federal funding, the NIH has been given a right to make the work publicly available in PubMed Central.

- National Heart, Lung, and Blood Institute (NHLBI), NIH, contract nos. 75N92022D00001, 75N92022D00002, 75N92022D00003, 75N92022D00004, and 75N92022D00005 (for the ARIC study) and in part by grant R01 HL134320.
- NIH grants R01DK124399 (to MEG, JC), K24HL155861 (to MEG), and R01CA267977 (to AEP).
- National Institute on Aging Intramural Research Program (to KW).
- German Research Foundation (DFG) Project-ID 530592017 (SCHL 2292/3–1) and Germany‘s Excellence Strategy (CIBSS – EXC-2189 – Project-ID 390939984).

## Supplementary Material

ICMJE disclosure forms

Supplemental tables 1-2

Supplemental tables 3-5

Supporting data values

## Figures and Tables

**Table 1:**
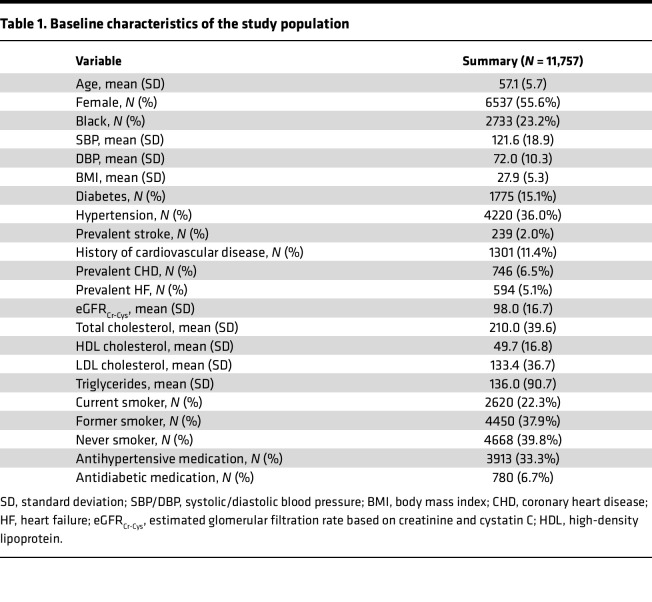
Baseline characteristics of the study population

**Table 2:**
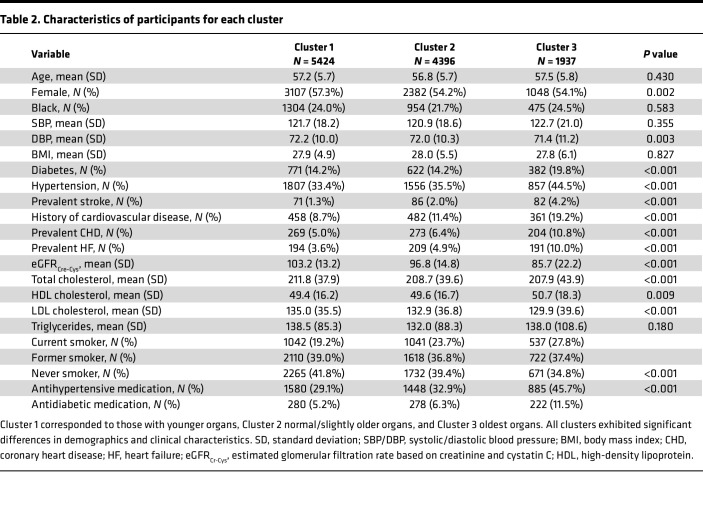
Characteristics of participants for each cluster

## Version 1 12/08/2025

Electronic publication
